# Photonic slide rule with metasurfaces

**DOI:** 10.1038/s41377-022-00765-0

**Published:** 2022-03-29

**Authors:** Feilong Yu, Jin Chen, Lujun Huang, Zengyue Zhao, Jiuxu Wang, Rong Jin, Jian Chen, Jian Wang, Andrey E. Miroshnichenko, Tianxin Li, Guanhai Li, Xiaoshuang Chen, Wei Lu

**Affiliations:** 1 State Key Laboratory of Infrared Physics, Shanghai Institute of Technical Physics, Chinese Academy of Sciences, 500 Yu Tian Road, 200083 Shanghai, China; 2 Hangzhou Institute for Advanced Study, University of Chinese Academy of Sciences, No.1 SubLane Xiangshan, 310024 Hangzhou, China; 3 Shanghai Research Center for Quantum Sciences, 99 Xiupu Road, 201315 Shanghai, China; 4 University of Chinese Academy of Science, No.19 Yuquan Road, 100049 Beijing, China; 5 School of Engineering and Information Technology, University of New South Wales, Canberra, 2602 Australia

**Keywords:** Metamaterials, Sub-wavelength optics, Nanophotonics and plasmonics

## Abstract

As an elementary particle, a photon that carries information in frequency, polarization, phase, and amplitude, plays a crucial role in modern science and technology. However, how to retrieve the full information of unknown photons in an ultracompact manner over broad bandwidth remains a challenging task with growing importance. Here, we demonstrate a versatile photonic slide rule based on an all-silicon metasurface that enables us to reconstruct incident photons’ frequency and polarization state. The underlying mechanism relies on the coherent interactions of frequency-driven phase diagrams which rotate at various angular velocities within broad bandwidth. The rotation direction and speed are determined by the topological charge and phase dispersion. Specifically, our metasurface leverages both achromatically focusing and azimuthally evolving phases with topological charges +1 and −1 to ensure the confocal annular intensity distributions. The combination of geometric phase and interference holography allows the joint manipulations of two distinct group delay coverages to realize angle-resolved in-pair spots in a transverse manner- a behavior that would disperse along longitudinal direction in conventional implementations. The spin-orbital coupling between the incident photons and vortex phases provides routing for the simultaneous identification of the photons’ frequency and circular polarization state through recognizing the spots’ locations. Our work provides an analog of the conventional slide rule to flexibly characterize the photons in an ultracompact and multifunctional way and may find applications in integrated optical circuits or pocketable devices.

## Abstract (web summary)

Metasurface-based photonic slide rule enables the simultaneous resolving of unknown photons’ wavelength and polarization state in mid-infrared.

## Graphical abstract

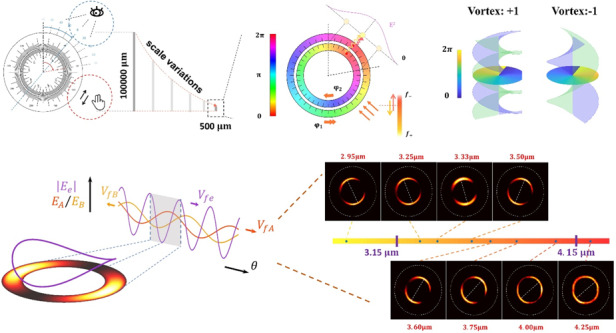

## Introduction

Photons carry multidimensional information in the forms of frequency, amplitude, phase, and polarization dimensions. Conventional scenarios to decouple the information from photons always require multiple and cascading optical elements, for example, discrete filters or polarizers which are rotated or spatially multiplexed to identify the spectral or polarization information^1–3^, narrowband tunable filters^4^, complex grating-based dispersive or Fourier transform-based spectrometers^5–7^. They are always bulky, especially along the longitudinal direction. For grating or Fourier spectrometers, size, mass, and power consumption are non-negligible. Mid-wavelength infrared (MWIR) is a unique regime with various potential applications in fingerprint detections since the vibrational absorption wavelengths of many molecules locate in this regime. Besides, it is also one of three atmospheric transmission windows in infrared, which show many significant possibilities in low-light-level night vision and free-space communications. It plays an indispensable role in aeronautics and astronautics applications.

The emergence of metasurface provides a powerful and flexible platform to independently manipulate the multiple dimensions of photons in subwavelength scale^8,9^, and has led to many interesting applications, such as super-resolution imaging^10–13^, multicolor holography^14–17^, and arbitrary beam generations^18–20^. The interleaved arrangement of metaatoms allows the metadevice to accomplish much more complex and complicated functionalities such as vectorial holography, optical data storage, and communications, which is inaccessible with a single discrete optical element^21–23^. To date, most of the studies focus on the control of amplitude, phase, and polarization of the given photons, but little effort was devoted to the characterization of unknown photons. The only several works for wavelength resolving of photons with metasurface are inherent to the extension or multiplexing of the functions, which are particularly restricted to the longitudinal dispersion^24–26^. Off-axis-focusing metalens that combines both focusing and dispersive functions can efficiently send light to larger angles^27^. However, such off-axis-focusing metadevice is usually limited by various aberrations like astigmatism and field curvature. Besides, a metadevice that can focus different incident wavelengths within a given bandwidth to three distinct spots is numerically presented to circumvent the spatial multiplexing constraint^28^. This metadevice could potentially replace the Bayer color filters which are widely used in color cameras. However, the platform is composed of complicated TiO_2_ nanostructures embedded in the SiO_2_ matrix, making fabrication of such a device extremely challenging. To our knowledge, metadevices that enable simultaneous resolving frequency and polarization state have not been reported yet. For applications that have strict restrictions on the volume or weight, such as space or atmosphere detections carried on the airplanes or satellites, for example, lab-on-a-chip, in situ or even in vitro characterizations, resolving and manipulating the photons in a versatile and compact manner is a challenging task yet of growing importance. Therefore, it is of urgent demand to simultaneously acquire multiple dimensional information of unknown photons, including both frequency and polarization state.

Inspired by conventional slide rules, which embed the computation rules into the inherent evolutions of physical parameters^29^, we present a new concept-photonic slide rule and demonstrate the prototype to characterize incident photons’ frequency and polarization state. With an all-silicon metasurface design, different wavelengths and polarization states correspond to different phase profiles, thus resulting in the angle-resolved focusing spots in the far-field. Two distinct groups of metaatoms are designed to have different vortex beam carriers and phase dispersions. The angle-resolved interference pattern in the far-field of two groups of metaatoms provides an easy-to-access way to retrieve the wavelength and the state of polarization. As an analog, we successfully migrate the arithmetic logic of dispersive phases to the inherent variation of photonic dimensions-frequency and polarization with the all-silicon metasurface.

## Results

**Fig. 1:**
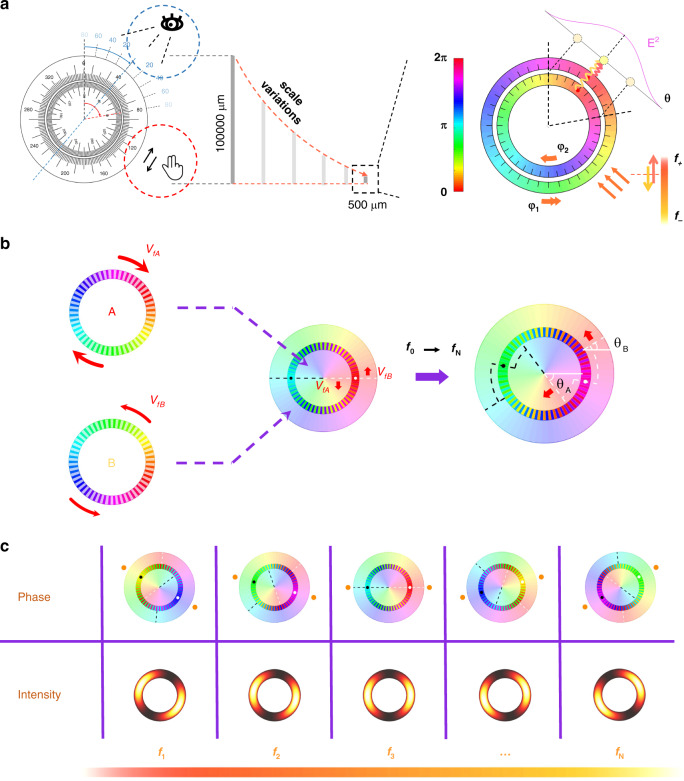
Concept of the metasurface design. **a** The conventional slide rule (left) is driven by mechanical rotation and read out visually with an easy-to-use size. As an analog, an ultracompact prototype of the photonic counterpart (right) based on the metasurface platform is shown. The metadevice is designed with the incident frequency and polarization state function with varying phases. Correspondingly, angle-resolved interfered spots are observed in the far-field. **b** The schematic of the interference evolution of two phase groups at different frequencies. The interference pattern changes with the incident frequency. **c** The expected overall performance of the metadevice at the sampling frequencies. The interfered annuluses rotate with the frequency, thus providing a route to recognize the wavelength of incident photons.

It is not straightforward to transplant the mechanically-tuning conventional slide to characterize the wavelength and polarization of photons in an ultracompact configuration as shown in Fig. 1a. To solve this, we choose two frequency-dependent phase profiles as the carrier of the unknown photons, and the focused interfered annular intensities in the far-field as the output to retrieve the exact wavelength and the state of polarization. Detailed analog can be found in Supplementary Note 1.

Next, in Fig. 1b we will show how the two-phase profiles are designed to experience opposite rotations with different speeds as a function of frequency: one rotates clockwise, and the other rotates anti-clockwise. The middle panel represents the alternating combination of the inner and outer phases. Once a prescribed phase at the starting frequency point is set, the inner and outer phase distributions are also determined within the whole operation bandwidth. When the frequency varies from *f*_0_ to *f*_*N*_, the vortex phase profile in the inner ring rotates −*θ*_*A*_ degree. Accordingly, the phase profile in the outer ring rotates +*θ*_*B*_ degree due to the different properties of the metaatoms. The relative displacement between the two phase profiles leads to the variation of far-field intensity distributions. Consequently, two bright spots located at the angles of (−*θ*_*A*_ + *θ*_*B*_)/2 and (−*θ*_*A*_ + *θ*_*B*_)/2+π, respectively. The mapping relationship between the incident frequency, corresponding phase profiles, and focused far-field intensity distributions allow the frequency recognition by reading the angle of interference spots. Figure 1c shows the schematic rotating angular interference spots with varying frequencies.

**Fig. 2:**
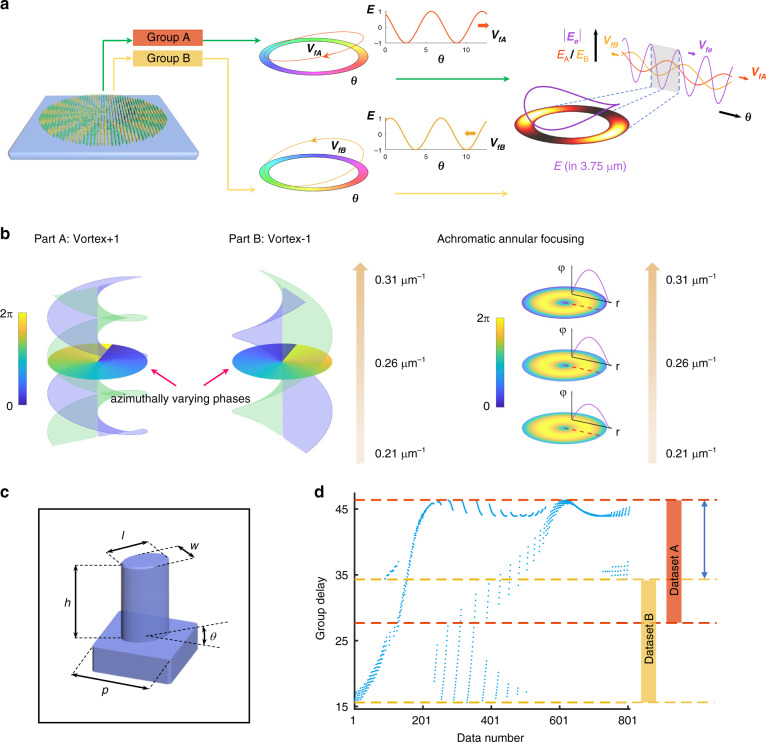
Implementation of the photonic slide rule with metasurface. **a** The metadevice comprises interleaved metaatoms of Group A and Group B, which generate two opposite phase vortices driven by the incident frequency. The linear plots along the phase annuluses are illustrated. The interference holography generates a moving symmetric annulus with intensity distribution indicated as the purple curve on the right. As a representative, intensity distribution at wavelength 3.75 μm is for demonstration. **b** The metadevice design can be decoupled to two parts: vortex generation and achromatic annular focusing. Both phase profiles are dependent on the frequency. In the vortex beam design, the phases rotate in opposite directions and at distinct angular velocities with different topological charges. The achromatic annular focusing design allows the enhanced intensity distributions and ensures the confocal spots at different frequencies. **c** Schematic of the metaatom made in a pure silicon wafer. The metaatom period is 1.7 μm, and the height is 7 μm. The width *w*, length *l*, and rotation angle *θ* are swept to construct the phase and dispersion control database. **d** The group delay coverages of the Group A and Group B metaatoms selected from the metaatom database. The data number represents the metaatom number with filtering criteria in the achromatic design of Supplementary Note 2.

After understanding how photonic slide rule works, we implement such a device with all-silicon metasurface. Because two sets of phase profiles that rotate at either clockwise or anti-clockwise directions are required, we need to utilize two interleaved groups of metaatoms, as shown in Fig. 2a. Each group of metaatoms carries distinct phase and phase dispersion profiles. Besides, two groups of metaatoms are respectively endowed with vortex phases of topological charge +1 and −1 since different frequency-dependent phase responses are required. Therefore, the phase profiles rotate in opposite directions as the incident frequency changes. When the two groups of metaatoms interfere in the far-field, the frequency-dependent and angle-resolved intensity distributions would be observed on the focal plane. Without loss of generality, we choose a wavelength of 3.75 μm for concept illustration, as shown in Fig. 2a. The normalized distributions in the far-field along the annulus follows the equation

1 $$\left| {E_e\left( {f,\theta } \right)} \right| = {\rm{abs}}\left( {{\rm{e}}^{i\cdot \left( {\frac{\theta }{{360}}\cdot 2\pi + \varphi _{A0} + {\it{V}}_{fA}\cdot \left( {f \,-\, f_0} \right)} \right)} + {\rm{e}}^{i\cdot \left( { - \frac{\theta }{{360}}\cdot 2\pi + \varphi _{B0} + V_{fB}\cdot \left( {f \,-\, f_0} \right)} \right)}} \right)$$

where *φ*_*A*0_ and *φ*_*B*0_ are the initial phases of the vortexes of A and B at *f*_0_, respectively. θ represents the azimuthal angle starting from the positive *x*-axis. *V*_*fA*_ and *V*_*fB*_ determine the rotation velocities of the phase profiles.

To construct an ultracompact metadevice, the focal length should be designed with a finite value. This implies that the annular intensities on the focal plane would be further enhanced compared with the case of an infinitely large focal distance. Besides, in order to ensure the same focusing spots profiles, an annular focusing phase profile with radius r_0_ is adopted

2 $${\upvarphi}\left( {r,\lambda } \right) = \left[ {2\pi \cdot \left( {F - \sqrt {(r - r_0)^2 + F^2} } \right)} \right] \cdot \frac{1}{\lambda } + \varphi _f\left( \lambda \right)$$

where *F* is the focal length and *φ*_*f*_ is the initial phases at different wavelengths. Without the annular focusing, the focusing spots are different for different wavelengths due to the diffraction-limited full-widths at half-maximum of the spots are dependent on the wavelength.

To construct the metadevice, the planar geometric parameters –length, width, and rotation angle of the birefringent all-silicon metaatom shown in Fig. 2c are swept to build the database of phase and phase dispersion. The selection of silicon material has three merits compared with other materials: high refractive index, which can provide broad dispersion coverage, most mature manufacturing processing, which facilities the fabrication, and transparent window in the mid-wavelength infrared due to its lossless nature. Corresponding metaatoms which are chosen from the database to construct the metadevice are shown in Fig. 2d. They are sorted according to the provided values of group delay. Points within orange and yellow shadows represent the two groups of metaatoms that are selected, respectively. The group delay coverage provided by the metaatoms is from 14.88 to 49.33, which is about 5.5 times 2π. Here, we set Group A’s starting group delay value as 49.33 and Group B as 33.60, respectively. The width of the double-headed blue arrow determines the rotating angle range of the two groups in which the maximum group delay is set as the benchmark. This group delay difference brings about the opposite and distinct angular rotation speeds in Fig. 2b. In Supplementary Note 2 the influence of group delay coverage difference on the rotation angle is discussed in detail.

Therefore, as shown in Fig. 2b the metasurface design can be divided into vortex beam generation and achromatic annular focusing. The former generates two opposite vortices with topological numbers +1 and −1 for the beam carriers, and the latter is to achieve achromatic annular focusing on the focal plane. Here, it’s worth noting that the achromatic design is necessary since there is a natural longitudinal aberration due to the phase dispersion in the focusing equation. The achromatic design ensures the same focal length for all the wavelengths within the operation bandwidth. The design details of achromatic annular focusing and the control of the rotation speeds and directions can be found in Supplementary Note 2. We also want to mention that the azimuthally varying phases other than a common unified phase are applied to achieve the high-intensity contrast on the focal plane. Details on the vortex selection and the effects of different topological charge orders on the characterization of photons can be found in Supplementary Note 3.

**Fig. 3:**
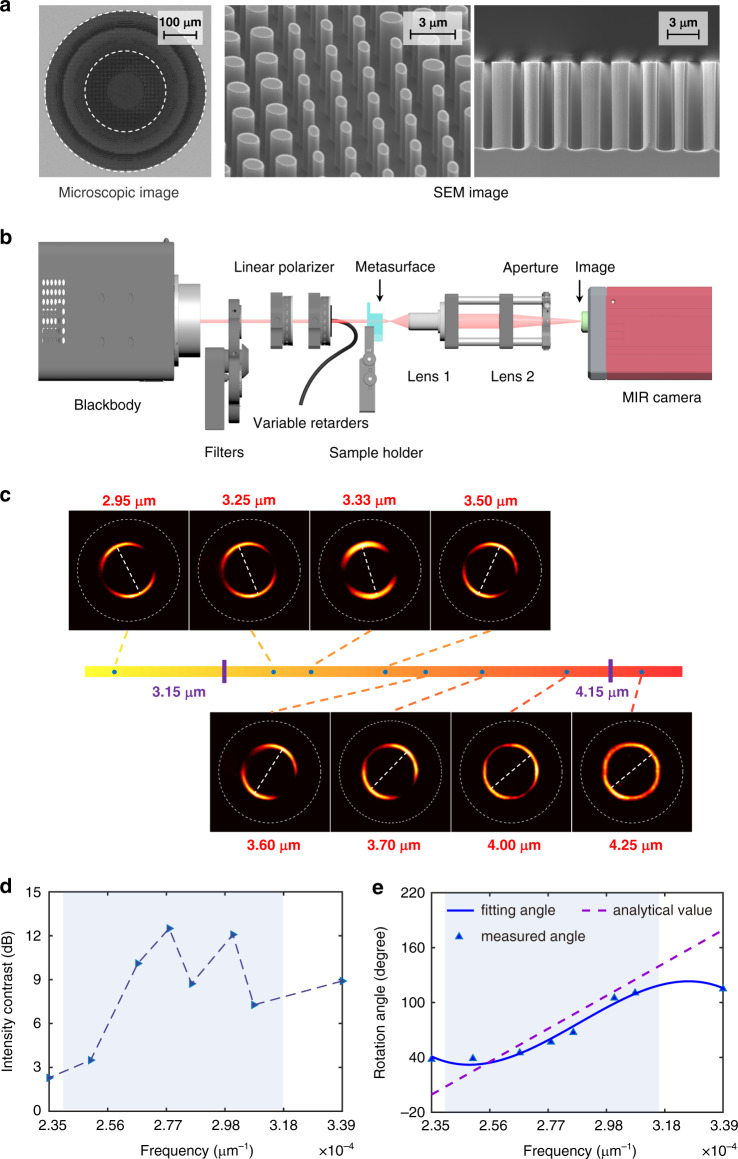
Fabrication and characterization of the metadevice. **a** Optical and scanning electron microscopes (SEM) images of the fabricated all-Si metasurface from different angles. **b** The experimental measurement setup for performance characterization. Blackbody, a linear polarizer, and liquid crystal retarder are used to generate collimated and the broadband incidence with selected polarization states. A series of commercial bandpass filters with 50–250 nm bandwidths are adopted to produce monochromatic light. The mid-wavelength infrared camera captures the interfered patterns in the far-field cooled at about 80 K. **c** The captured images at different frequencies on the same focal plane with left circular light incidence. The annular intensity distribution rotates with the incident frequency. **d** The intensity contrasts at different frequencies. The values are on a logarithmic scale. **e** The mapping relationship between the rotation angle of the interfered spots and the frequency. The analytical values are also plotted for comparison as indicated by the purple dashed line. The gray zones highlighted in (**d**) and (**e**) denote the designed operation bandwidth from 3150 nm to 4150 nm. The blue background in (**d**) and (**e**) represents the designed operation bandwidth.

We then moved to the experimental demonstration of such metadevice. For the sake of simplicity, we first consider a simple function that could resolve the frequency. Figure 3a shows the optical and SEM images of the fabricated metadevice with a diameter of 500 μm. The tilted and cross-section images of the zoom-in metaatoms illustrate the excellent homogenous fabrications, especially for metaatoms with different aspect ratios. The white circle represents the profile of the annular focusing spot in the far-field. The radius r_0_ is 125 μm and focal length F is 650 μm. To characterize the metadevice, we built a homemade measurement system shown in Fig. 3b. Details on the fabrication and experiment setup can be found in the “**Method**” section. Lens 1, lens 2 and the aperture are fixed without any movement during the measurement. We did not introduce any artificial adjustment on the optical elements and the sample, but only controlled the filter electrically to select bandpass filters.

The measured intensity distributions on the designed focal plane are captured for different frequencies across the operation bandwidth as shown in Fig. 3c. The focused annular spots rotate when increasing the incident frequency. We plot the intensity contrasts along annuluses at different wavelengths to accurately determine the angles in Fig. 3d. More specifically, the annuluses’ intensities are first extracted before the line fitting method retrieves the maximum intensity positions. The positions are in a consistent one-to-one match to the rotation angles. This method allows us to determine the angle difference for two known neighboring wavelengths accurately. The intensity contrast is defined as the ratio of the strongest light intensity and the weakest on the annuluses in the logarithmic scale. The interfered spots have high contrasts larger than 5 dB within the designed bandwidth. Despite the contrast declines at the ends of operation bandwidth, the performance is still good enough to recognize the rotation angles clearly.

To evaluate the performance of metadevice design, we plot the theoretically predicted and experimentally measured angles as a function of frequency in Fig. 3e for direct comparison with the theoretical values derived in Supplementary Note 2. It can be seen that the experimental results are in excellent agreement with theoretical ones. Beyond the designed wavelength range, the measured angles tend to deviate slightly from the designed ones. This phenomenon can be attributed to the fact that the dispersion curves of the metaatoms are not strictly linear at wavelengths out of the designed bandwidth. Since the deviation trend is consistent for all the metaatoms, the minor misregistration of the rotation angle has little influence on the far-field interference patterns. Therefore, over the entire bandwidth, the interference spots maintain continuous rotations. Related simulation results can be found in Supplementary Note 4.

**Fig. 4:**
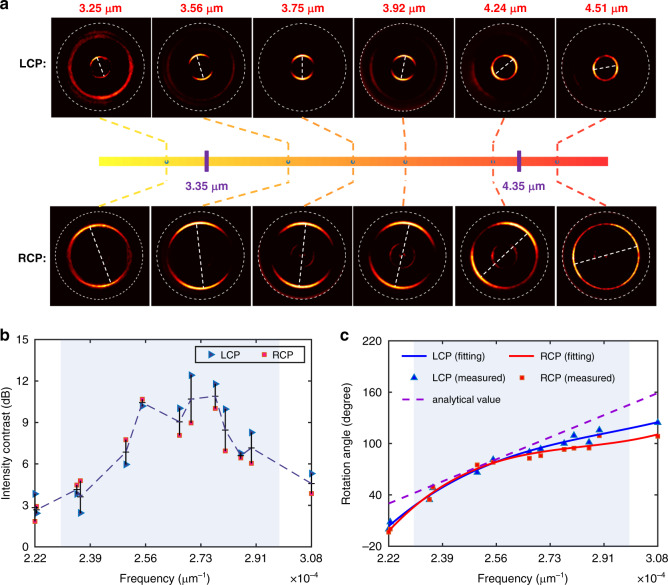
Simultaneous characterization of frequency and polarization state. **a** The captured images on the focal plane at different frequencies under left and right circular polarization incidence. The inner and outer rings are respectively lightened as a function of the incident polarization state. **b** Intensity contrasts of the measured results at different frequencies. The values are on a logarithmic scale. The blue points represent the cases under the left circular polarization and the red ones for the right circular polarization state. **c** Relationship between the rotation angle of interfered spots and the incident frequency. The dashed purple line represents the analytical values. The blue background in (**b**) and (**c**) represents the designed operation bandwidth. LCP: left-handed circular polarization, RCP: right-handed circular polarization.

Since two groups of metaatoms with distinct dispersive properties like Fig. 3 are used to resolve the wavelength of left circular polarized incidence, another two groups of metaatoms which correspond to its orthogonal counterpart -right circular polarized light are interleaved to generate another focused annulus. With this handling, circular polarization states can be distinguished. We fabricate such a photonic slide rule based on the all-silicon metasurface and characterize it with the same measurement system shown in Fig. 3b. Figure 4a shows the far-field intensity distribution when considering incident photonics with different frequencies and circular polarization. Similar to Fig. 3, the rotated angle of far-field intensity profiles could differentiate the frequency of incident photonics. Additionally, the circular polarization information could be directly retrieved from the position of focusing spot. The inner annulus represents the left-handed circular polarization state cases, while the outer one denotes the right circular polarization state. The inner radius is 75 μm and outer radius is 200 μm. The focal length is 350 μm. The excellent performances are further confirmed in Fig. 4b by extracting the intensity contrast from Fig. 4a. All the measured contrasts within the designed bandwidth are larger than 3 dB. Besides, we retrieve the rotation angles at different frequencies and illustrate them in Fig. 4c. Excellent agreement is achieved by comparing the theoretical values and measured ones for both circular polarization states. It’s worth mentioning that the method is not limited to characterizing the circular polarization states. The most general case-elliptical polarization can also be resolved. Details can be found in Supplementary Note 5.

**Fig. 5:**
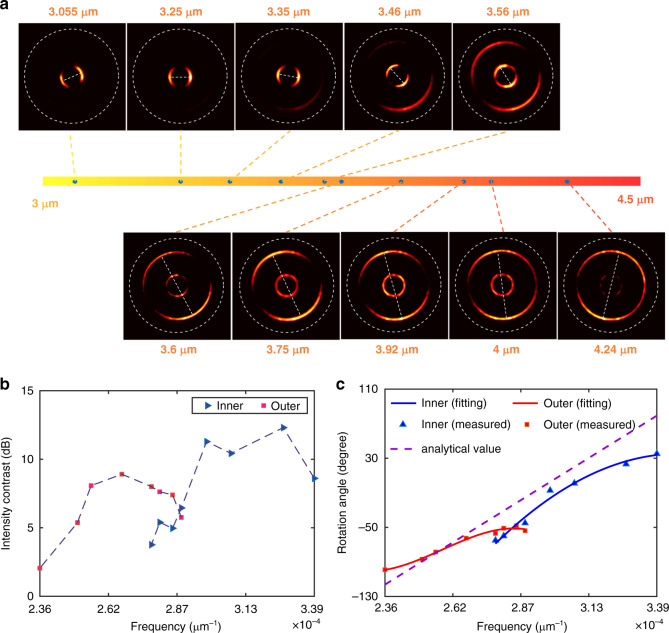
Experimental results of the metadevice for wavelength extension. **a** The captured images on the same focal plane at different frequencies for left circular polarization incidence. The inner and outer rings are lightened with an incidence at wavelength bands of 3000 nm −3600 nm and 3600 nm −4300 nm, respectively. **b** Intensity contrasts of the measured results as a function of frequency for different operation bands. The values are on a logarithmic scale. **c** Relationship between the rotation angle of interfered spots and the incident frequency over two designed wavelength ranges. The dashed purple line represents the analytical values.

Although the operation bandwidth of the metadevices works in the range from 3350 nm to 4350 nm due to the inherent dispersion property of selected metaatoms, in fact, their operation bandwidth can be further extended through spatially multiplexing with one more annulus. For example, in Fig. 5a we show the metadevice with inner and outer annuluses designed for wavelength ranges 3000 nm −3600 nm and 3600 nm −4300 nm, respectively. The inner radius is 62.5 μm and outer radius is 187.5 μm. The focal length F is 250 μm. In Fig. 5b, among the bandwidth 3000 nm −3600 nm, the inner annulus rotates clockwise with intensity contrasts larger than 5 dB as the wavelength increases. Compared with the inner one, the outer annulus shows superior performance from the observation of line fitting in Fig. 5c. The minor deviations from the analytical values can be attributed to the fact that the metaatoms selected from the database for phase and phase dispersion control are not perfectly the same as design. Some metaatoms are even multiplexed for the generation of both annuluses, leading to unexpected intensity distributions along the annuluses. However, there is no obvious interference between the two annuluses. The experimental results indicate that the superposition of multifunctions has little crosstalk between each other. Therefore, more complex functions may be achieved with a dedicated design.

## Discussion

Inspired by the conventional slide rule, we present an entirely new concept-photonic slide rule based on the all-silicon metasurface to successfully demonstrate the flexible characterization of the multiple dimensions of unknown photons in an ultracompact and multifunctional way in MWIR. In particular, we illustrate two metadevices that are capable of resolving the frequency separately or characterizing the frequency and polarization state simultaneously. Besides, the operation bandwidth extension is also implemented with the same method through merely adding one more annulus. The experimental results agree well with the design within the operation bandwidth.

Actually, several metrics accompany the metadevice: operation bandwidth, wavelength resolution, polarization state, sample size, working distance, operation speed, mass, power consumption and so on. In the following, we mainly discuss the operation mode, wavelength resolution, and complete polarization state resolving of the metadevice.

The metadevices can operate in two conditions according to the working distance: infinite and finite modes. If the focusing phase profile is not imparted to the metaatoms or the focusing distance is set as infinite, the metadevices would work in the infinite mode. Only two fixed metaatoms are required to implement the interference effect by adjusting the geometric metaatoms’ rotation angles in this mode. However, this scenario inevitably imposes a severe restriction on the operation distance along the longitudinal direction since it has an infinitely large working distance, which fundamentally contradicts the initial motivation to realize ultracompact and adjustable operation distance metadevices. If the focusing phase profiles are imparted to the metaatoms, the metadevices will operate in the finite mode. Phase and phase dispersion should be simultaneously satisfied to ensure that the focusings at different wavelengths are all in the same focal plane. Otherwise, the focal lengths are distinct for different wavelengths. Therefore, the phase and its dispersion are manipulated to have a constant focal plane within operation bandwidth. The propagation phase is adopted to satisfy the simultaneous phase and phase dispersion requirements through tuning the geometric parameters. Therefore, the width, length, and rotation angle are swept at a fixed height to build up the phase and group delay database. One thing worth noting is that the adoption of focusing phase profiles also enhances the intensity and improves the imaging contrast.

This work extracts the intensity distributions around the annuluses before the line fitting method is applied to retrieve the maximum intensity positions, which are in a consistent one-to-one mapping between the horizontal positions and the rotation angles. This method allows us to determine the angle difference for two known neighboring wavelengths accurately. This approach is also feasible for wavelength resolution calibration since there are some minor deviations from the desired angles at the ends of the operation bandwidth.

With the metadevice design, the wavelength resolution can be evaluated through the following expression:

3 $$\theta = \frac{1}{2} \cdot \left( {Q_2 - Q_1} \right) \cdot \frac{f}{{f_0}}$$

where θ is reading angle, *Q*_1_ and *Q*_2_ are the normalized group delay coverages of Group A and Group B metaatoms with center frequency *f*_0_, respectively. *f*_0_ = 2.642 × 10^−4^ nm^−1^ (3785 nm), *Q*_1_ = 33.6, *Q*_2_ = 49.3. The derivative of Eq. (3) is

4 $$\frac{{{\rm{d}}\theta }}{{{\rm{d}}f}} = \frac{{Q_2 - Q_1}}{{2f_0}} = 2.97 \times 10^4{{{\mathrm{rad}}}} \, {{{\mathrm{nm}}}}$$

For an example, we choose two wavelengths 3780 nm and 3790 nm as demonstration to evaluate the angular difference in the far-field focused annulus. The angle difference can be calculated as ${\Delta}\theta = \frac{{{\rm{d}}\theta }}{{{\rm{d}}f}} \times {\Delta}f = 2.97 \times 10^4 \times \left( {\frac{1}{{3780}} - \frac{1}{{3790}}} \right) = 0.0207$ rad. On the annular focal plane, the arc length corresponding to this angle difference is ∆θ × 125 μm = 2.5875 μm. Here, 125 μm is the radius of the focusing annulus. It doesn’t mean that the resolution of the metadevice is 10 nm. Due to the restriction of high-performance MWIR laser and detector, we choose discrete bandpass filters to characterize the metadevice on the wavelength resolving property.

In the measurement series of discrete optical filters whose bandwidths change from 50 nm to 250 nm are chosen to evaluate the wavelength resolution. The bandwidth of 50 nm as measured using discrete filters has already met the requirements of hyperspectral applications like vapor or carbon dioxide detections in the mid-wavelength infrared. Obviously, 50 nm is far from the wavelength resolution of the metadevice. In practical implementation, the wavelength resolution can be further improved by enlarging the metadevice size or adopting optimization algorithms like those in computational spectral reconstruction–based spectrometers, where complex algorithms are used to approximate or reconstruct the incident light spectrum^30–32^.

The metadevice is still feasible, with three annuluses corresponding to three distinct polarization bases as the complete resolution of any polarization state. In this way, there are three focusing annuluses and maximum intensities to characterize any polarization state on the Poincare sphere. The polarization state can be uniquely determined by solving Stokes parameters’ equations. In Supplementary Note 5, resolving the most general case-elliptical polarization is discussed.

The corresponding scenarios would be much more complicated as to the incident photons with complex information. Multiple spots would occur along the annulus for multiple wavelengths with the same polarization. A multiple line fitting method will be adopted to reconstruct the wavelengths in this condition. As to the case of multiple wavelengths with different polarizations, at least three annuluses that correspond to other polarization bases are required to completely resolve the wavelength and polarization state. Therefore, not only the line fitting method is necessary, the equations as a function of the Stokes parameters also need to be solved. However, the fittings would be much more complicated over a continuous wavelength band. The postprocessing will dominate the measurement accuracy through our metadevice, which could capture the spectrum and polarization information of incident photons. It may require lots of complicated calculations before all the spectra and polarization information can be extracted. Here, we want to mention that the wavelength and polarization calibrations would increase the resolving accuracy.

As to the operation speed of the metadevice, we define the operation speed as the reverse of the time during which incident photons are focused from the metadevice to the focal plane and captured by the photodetector. Compared with the time spent integrating of photon-generated carriers in the photoelectric detector to generate a frame of the image, the time taken to generate focused spots from the metadevice to the focal plane is negligible. In our measurement, the integration time of the photodetector is set as 100 μs (minimum 1.1 μs). Our homemade MCT detector’s maximum frame per second (fps) can reach 1000 Hz. It’s worth noting that the integration time of the photothermal detector is much longer which is tens or hundreds of milliseconds, for example, the fps of state-of-the-art T860 from FLIR System, Inc. is 30 Hz. Though the time for photos crossing the distance from the metadevice to the focal plane is 1.67 × 10^−12 ^s (distance 500 μm, light speed 3 × 10^8 ^m/s), compared with those configurations with rotated optical elements, the operation speed is much faster since the switching time of electrically controlled rotator is tens or hundreds of milliseconds. For example, the minimum switching time of motorized filter wheel FW103H in Thorlabs Inc. is about 55 ms.

The designed metadevice has the potential to characterize broadband spectrum through postprocessing the captured angle-resolved intensities because different wavelengths would be projected to different positions on the far-field focal plane. Besides, the metadevice here can resolve the state of polarization. It’s worth noting that the dispersive or Fourier spectrometers are capable of achieve broadband spectrum with high resolution simultaneously. For grating-based dispersive spectrometers, they operate with propagation distances about tens of centimeters. For example, the distance of iHR550 from Horiba Inc. is 550 mm. Fourier transform-based spectrometers like Vertex 80 V from Bruker Inc. spend about 1 s or more on moving the mirrors to have interference for the wavelength resolution of 1 meV in MWIR (wavelength resolution is about 12 nm at center wavelength 4 μm).

As to the mass of the metadevices, the maximum size we fabricated with a monocrystalline silicon wafer is 500 μm × 500 μm. The thickness of the substrate is 500 μm. With the density of silicon 2.3 g cm^−3^, the weight of metadevice is 2.88 × 10^−4^ g, much less than the spectroscopical elements. Since the metadevice is passive, no power supply is required. This is superior to those needing electrically controlled motors to switch the gratings, wheels, or adjust the movable mirrors.

The metadevice has little dependence on the operation environments since it is only one chip-like silicon piece which are less sensitive. Besides, the metadevice has the potential to be integrated with the detector since the fabrication of the all-silicon metadevice has excellent compatibility with the existing silicon-based MCT detector manufacturing technology. If the metadevice is successfully integrated in the detector, conventional requirements on the mechanical vibration for the onboard satellites or airplanes applications are not necessary anymore^33,34^.

In this work, we try to demonstrate a new metasurface design methodology and prototype to simultaneously resolve the wavelength and polarization state in MWIR with an ultracompact and lightweight configuration. The material platform, metaatom shape, thickness, and arrangement rules are not limited. The design can be easily and straightforwardly transplanted to other wavelengths by choosing proper material platforms and having size magnification or minification of metaatoms. The material platforms should be lossless, and the materials’ growth and fabrication should be accessible. It would be better if the materials were cheap and the manufacturing technology is mature, like silicon, gallium nitride, and titanium dioxide, which are extensively investigated in literatures. The fine adjustment of the metaatom size would also be necessary. For example, in the implementation of resolving unknown photons in near-infrared, the all-silicon platform or silicon-on-silica substrate is feasible since both silicon and silica in this regime are lossless and easy to be fabricated with current manufacturing technology. The birefringent metaatoms can also be adopted. As long as the required phase and phase dispersion control can be fulfilled, the functionality implemented in MWIR can also be accessed in near-infrared. The selection of birefringent metaatoms is because the shape gives us more freedom to adjust the phase by changing its geometries. Composite or freeform shapes would bring much more freedom, improve diffraction efficiency, and broaden the operation bandwidth. It’s also worth mentioning that the overall arrangement optimization of metaatoms with algorithms would also benefit the metadevice performance. We believe that our work may stimulate more applications in the relatively immature mid-infrared meta-optics.

## Materials and methods

### Sample fabrication

An aluminum (Al) film with a thickness of 100 nm is deposited on one side of the 4-inch double-polished silicon wafer with e-beam evaporation method before the Ma-N 2403 negative photoresist is coated and baked. Electron beam lithography (JBX-6300FS) at accelerating voltage 100 kV is adopted to generate the pattern. The metadevice is developed in 300-MIF solution and rinsed with deionized water before drying with a low-temperature development machine. Inductively coupled plasma is successively used for dry etching of the Al layer and silicon etching with different gases. The feature width of the metaatoms is 250 nm with a neighboring distance larger than 300 nm.

### Measurement setup

The experimental apparatus is composed of input light control part, sample alignment part, and imaging part. The blackbody serves as the broadband light source and a series of narrowband filters to generate illuminations at different frequencies. Linear polarizer and liquid crystal retarder (LCC1113-MIR) are used to engineer the polarization state. The imaging part is mainly composed of a 4 mm aspheric lens and a 25 mm lens. The images of the sample on the focal plane are captured using the MCT mid-wavelength infrared camera with 640 × 512 pixels.

## Supplementary information

Supplementary material
