# Supplementary material for: Photonic slide rule with metasurfaces
Source: Light Sci Appl. 2022 Mar 29;11:77. doi: 10.1038/s41377-022-00765-0 (PMC8964711; doi:10.1038/s41377-022-00765-0)
Supplement: Supplementary file 1 — Supplementary material [file 41377_2022_765_MOESM1_ESM.docx]

# Supplementary Information for

# Photonic Slide Rule with Metasurfaces

Feilong Yu ^1,2,3,4#^, Jin Chen ^1,2,3,4#^, Lujun Huang^5^, Zengyue Zhao^1,4^, Jiuxu Wang^1,4^, Rong Jin^1,4^, Jian Chen^1,4^, Jian Wang^1,4^, Andrey E Miroshnichenko^5^, Tianxin Li^1,4^, Guanhai Li^1,2,3,4^**^*^**, Xiaoshuang Chen^1,2,3,4^, and Wei Lu^1,2,3,4^

*^1^State Key Laboratory of Infrared Physics, Shanghai Institute of Technical Physics, Chinese Academy of Sciences, 500 Yu Tian Road, Shanghai, 200083, China*

*^2^Hangzhou Institute for Advanced Study, University of Chinese Academy of Sciences, No.1 SubLane Xiangshan, Hangzhou, 310024, China*

*^3^Shanghai Research Center for Quantum Sciences, 99 Xiupu Road, Shanghai, 201315, China*

*^4^University of Chinese Academy of Science, No.19 Yuquan Road, Beijing, 100049, China*

*^5^School of Engineering and Information Technology, University of New South Wales, Canberra, 2602, Australia*

# These authors contributed equally: Feilong Yu, Jin Chen.

* Correspondence: Guanhai Li (*ghli0120@mail.sitp.ac.cn)*

## Note 1. Design concept-from conventional slide rule to photonic slide rule

Through taking advantage of the flexible metasurface platform, we transplant the computational mechanism of conventional slide rule to the 'photonic’ counterparts and thus realize characterizations of multiple dimensions of photons. In this work, the following analogs are utilized to fulfill the requirements:

1. The spatial rotation of the circular slide rules is transformed to the rotation of the delicately designed phase profiles.
2. The driving quantity is passed from the external force to the dimension of incident photons like frequency and polarization state.
3. The direct visual readout is to converted to the angle-resolved intensity distributions.
   1. ***The way* to drive *the relative* rotation of phase profiles**


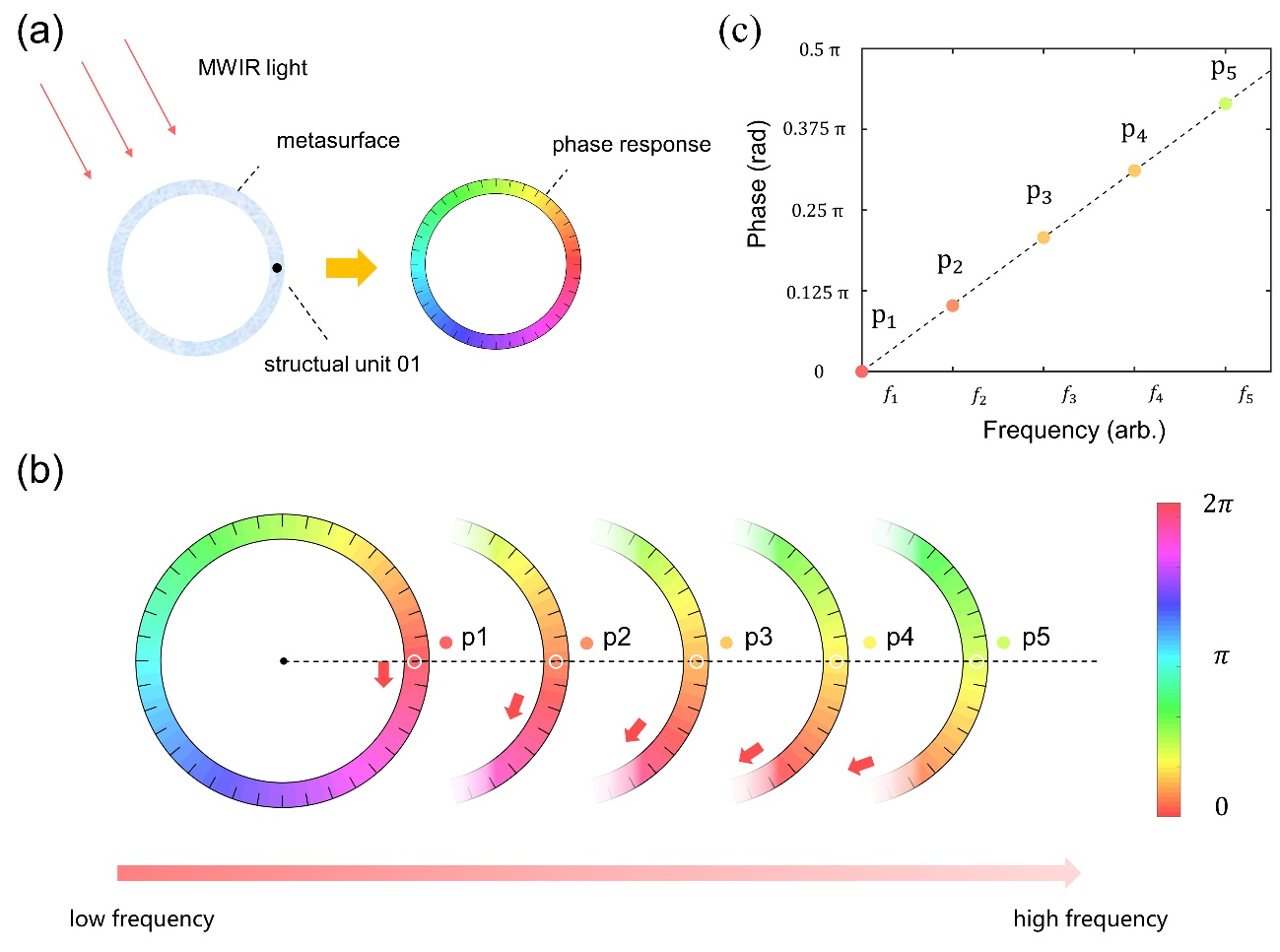


**Fig. S1 Schematic of the annular phase profile’s rotation driven by frequency. (a)** The metasurface is constructed to generate an annular phase profile when illuminated with MWIR light. **(b)** Schematic of the phase variations at different frequencies. **c** The phase curve as a function of frequency for a given point on the annulus.

Although the phase response of the metaatoms can be electrically controlled through changing the external voltage or capacitance, it is usually used in microwave or radiofrequency. In this paper, we propose an optical method to manipulate the phases. An optical vortex beam, whose phase profile varies with the angle, is inherently capable of mimicking the circular slide rule. The key is to find a controllable way in which the phase can be consistently rotated as a function of photons' dimensions. We take frequency as the driving quantity for the concept demonstration, as shown in **Fig. S1a**. The metasurface can be designed to generate annular phases with the illumination of mid-wavelength infrared. The black spot indicates the observation points. The phases can be accordingly varied as a function of frequency, as shown in **Fig. S1b**. Meanwhile, the selected observation point needs to be transplanted into the phases of [P1, P2, P3, P4, P5] as shown in **Fig. S1c.**

- 1. ***Readout of the calculation result***


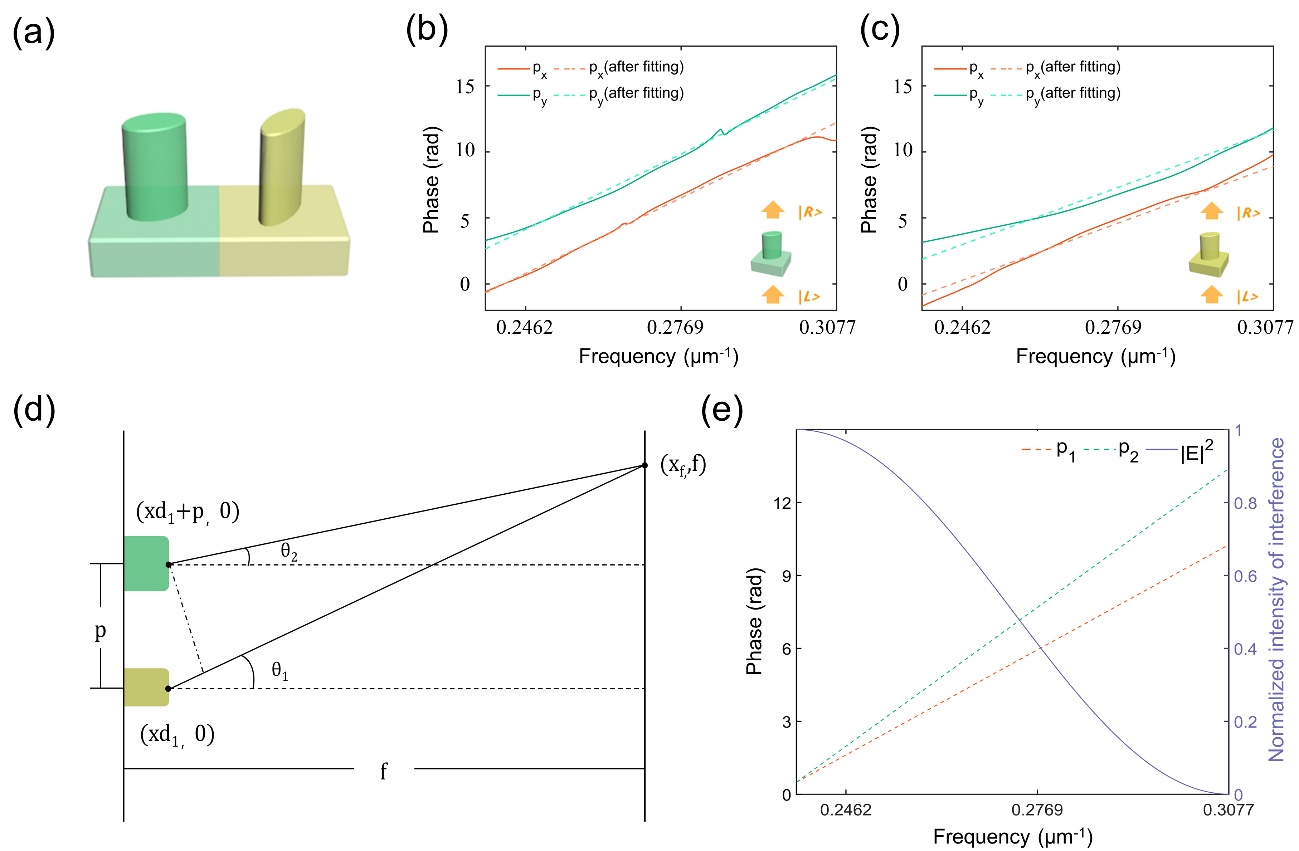


**Fig. S2 Schematic output of calculation result. (a)** Two representative metaatoms in green and yellow colors are selected for interference in the far-field. **(b)-(c)** The dispersion curves of two selected metaatoms which satisfy the geometric phase condition but have different dispersion slopes. (**d)** Schematic interference of the two metaatoms on the focal plane. *p* is the period, and *f* is the focal length. **(e)** The interfered intensity plots of the two selected metaatoms as a function of frequency.

Compared with traditional slide rules that eyes can directly read out, we need to find a new way to output the computing results for metasurface. Considering that phase is chosen as the operating carrier, the interference pattern in the far-field is used as the readout in this scenario. As shown in **Fig. S2a**, two groups of metaatoms are selected to realize the interference. In order to generate annular phase profiles, geometric phase structures are rotated to achieve 2π phase coverage. Here, it’s worth mentioning that it’s not enough when manipulating the phase dispersion only with geometric phase. **Figs. S2b** and **c** are the phase curves of two representative metaatoms for illustration. The phase difference between the long and short axes satisfies the geometric phase condition within the whole operating frequency range. **Fig. S4d** shows the schematic diagram of the interference of the two elements. The interference of the two metaatoms can be written as:

$E_{g}=E_{1}*exp\left[ i*(\varphi_{1}+\frac{2\pi}{\lambda}*\frac{f}{sin\theta_{1}}) \right]+E_{2}*exp\left[ i*(\varphi_{2}+\frac{2\pi}{\lambda}*\frac{f}{sin\theta_{2}}) \right]$ (S1)

where $\varphi_{1}$ and $\varphi_{2}$ are the initial phases, and $E_{1}$ and $E_{2}$ are polarization conversion efficiencies of two metaatoms, respectively.

Among them:

$\theta_{1}=arctan\left( \frac{x_{f}-{xd}_{1}}{f} \right)$ (S2)

$\theta_{2}=arctan\left( \frac{x_{f}-{xd}_{1}-zq}{f} \right)$ (S3)

When the period *p* is sufficiently small comparing to the focal length *f*, the interference can be expressed as:

$E_{g}=E_{1}*\exp\left( i*\varphi_{1} \right)+E_{2}*\exp\left( i*\varphi_{2} \right)$ (S4)

When two metaatoms with different dispersions interfere with each other, the far-field intensity changes with frequency, as indicated by the solid line in **Fig. S2(e)**.

By taking advantage of the new driving and readout methods based on metasurface, we are able to transplant the calculation logic of the conventional slide rule to the photonic slide rule.

## Note 2. Selections of metaatoms for dispersion manipulations

The adoption of the focusing function contributes to the functional performance of the metadevice, especially considering that it operates over a broad wavelength range. Besides, it is necessary to introduce the achromatic design to ensure all the interfered patterns locate on the same focal plane. This section will discuss how the achromatic design and vortex generation help underpin the photonic slide rule implementation.

1. ***Achromatic focusing design principales***


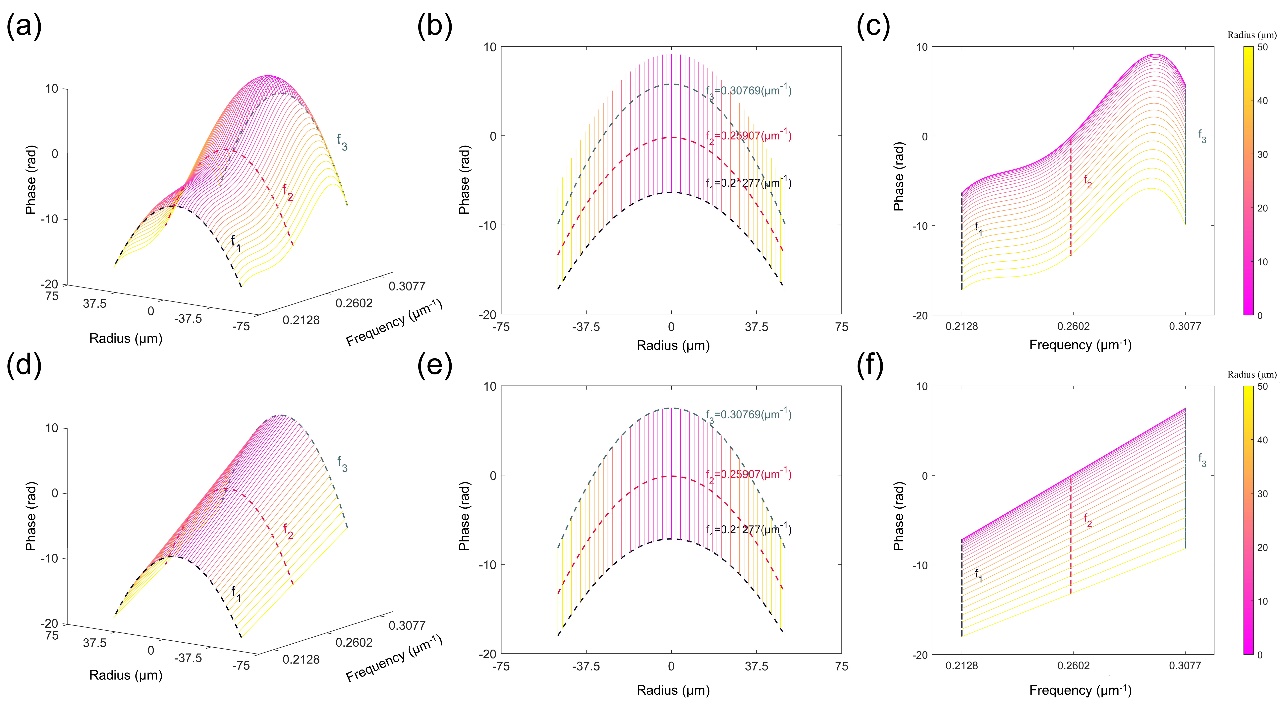


**Fig. S3 Schematic illustration of the achromatic concept.** The irregular dispersions of the metasurface for achromatic focusing from the views of **(a)** phase-frequency-coordinate, **(b)** phase-coordinate, and **c** phase-frequency. The schematic of the metasurface for achromatic focusing in linear dispersions are shown in **(d)** phase-frequency-coordinate view, **(e)** phase-coordinate view and **(f)** phase-frequency view, respectively.

Achromatic focusing is to achieve the same focusing effect without aberrations at different frequencies. Since the photons with different frequencies have different phase delays in air, the metasurface should have different optical responses at different frequencies to provide corresponding phase profiles. The lateral light field manipulation does not depend on the change of the basic phase $\varphi_{0}$, i.e., the phase profile can move freely up and down. **Figs. S3a-c** and **Figs. S3d-f** represent the achromatic phase profiles in irregular dispersions and linear dispersions, respectively. They have the same focal length and achromatic focusing effect since their phase profiles have the same shape for the designed frequencies. **Fig. S3c** and **Fig. S3f** show the corresponding phase-frequency dispersion curves that need to be selected out. Quadratic and cubic polynomial corrections are adopted for fittings. The dispersion curves of the metaatoms are chosen to be linear.

In the following, we illustrate the achromatic design process. From the general equation for focusing at a given wavelength:

$\varphi\left( r,\lambda\right)=\left[ 2\pi\cdot\left( F-\sqrt{r^{2}+F^{2}} \right) \right]\cdot\frac{1}{\lambda}+\varphi_{f}\left( \lambda\right)$ (S5)

For each metaatom, the first term on the right represents the phase requirement at wavelength λ. The latter term $\varphi_{f}\left( \lambda\right)$ corresponds to the arbitrary wavelength-dependent phase that does not affect the overall phase profiles at each wavelength. It can be rewritten in the linear form and thus **Eq. S5** can be modified as:

$$\varphi\left( r,\lambda\right)=\left[ 2\pi\cdot\left( F-\sqrt{r^{2}+F^{2}} \right) \right]\cdot\left( \frac{1}{\lambda}-\frac{1}{\lambda_{0}} \right)+\left[ 2\pi\cdot\left( F-\sqrt{r^{2}+F^{2}} \right) \right]\cdot\frac{1}{\lambda_{0}}+k\cdot\left( \frac{1}{\lambda}-\frac{1}{\lambda_{0}} \right)+\varphi_{0}$$

$=\left\{ \left[ 2\pi\cdot\left( F-\sqrt{r^{2}+F^{2}} \right)+k \right]\cdot\left( \frac{1}{\lambda}-\frac{1}{\lambda_{0}} \right) \right\}+\left\{ \left[ 2\pi\cdot\left( F-\sqrt{r^{2}+F^{2}} \right) \right]\cdot\frac{1}{\lambda_{0}}+\varphi_{0} \right\}$ (S6)

$F$ denotes the focal length, *k* is the first derivative of the additional phase, $\lambda_{0}$ is the selected central wavelength, $\varphi_{0}$ is the additional phase at the central wavelength and $r$ is the coordinate of the metaatom. The first derivative of the formula becomes $2\pi\cdot\left( F-\sqrt{r^{2}+F^{2}} \right)+k$, and the constant term of the formula is $\left[ 2\pi\cdot\left( F-\sqrt{r^{2}+F^{2}} \right) \right]\cdot\frac{1}{\lambda_{0}}+\varphi_{0}$. These two terms are the parameters that we need to extract from the dispersion curves. During the metasurface implementation, we need to select and arrange suitable metaatoms from the database to fulfill the required phase at the coordinates from $r_{min}$ to $r_{max}$. Since both phase terms vary with $r$, there is a limiting relationship between the first derivative and the constant term. That is to say when selecting different global initial values $k$ and$\varphi_{0}$, different selection ranges with varying conditions of restriction are provided. This will restrict the maximum device size that can be achieved. Therefore, we need to optimize the global initial values $k$ and$\varphi_{0}$ to enhance the metadevice performance.

Next, we will discuss how to choose the best global initial phase values. To simplify the selection process, we extract the relationship between the first-order differential and the second-order differential as:

$k_{d}=2\pi\cdot\left( F-\sqrt{r^{2}+F^{2}} \right)+k$ (S7)

$\varphi_{d}=\left[ 2\pi\cdot\left( F-\sqrt{r^{2}+F^{2}} \right) \right]\cdot\frac{1}{\lambda_{0}}+\varphi_{0}$ (S8)

where $k$, $\varphi_{0}$,$\lambda_{0}$ are the initial global values. With the **Eqs. S7** and **S8**,

$$k_{d}\cdot\frac{1}{\lambda_{0}}-\varphi_{d}=\left[ 2\pi\cdot\left( F-\sqrt{r^{2}+F^{2}} \right)+k \right]\cdot\frac{1}{\lambda_{0}}-\left\{ \left[ 2\pi\cdot\left( F-\sqrt{r^{2}+F^{2}} \right) \right]\cdot\frac{1}{\lambda_{0}}+\varphi_{0} \right\}$$

$=k\cdot\frac{1}{\lambda_{0}}-\varphi_{0}$ (S9)

$k\cdot\frac{1}{\lambda_{0}}-\varphi_{0}$ is also a constant and can be used as the rapid filtering criteria to extract the first derivative $k_{d}$ and the phase $\varphi_{d}$ at center wavelength$\lambda_{0}$. Under the condition that the metaatoms should provide the same value as $k_{d}\cdot1/{\lambda_{0}}-\varphi_{d},$ the metaatoms can be chosen. This makes it easy to directly identify the dataset that can provide the largest dispersion control range, as shown in **Fig. S4**.


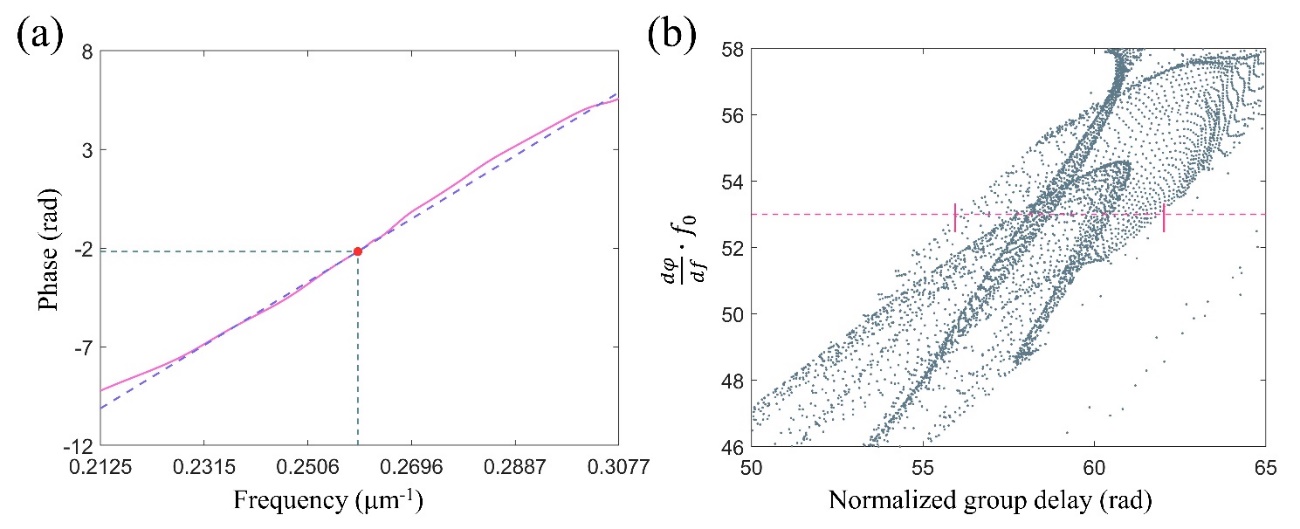


**Fig. S4 Schematic diagram of metaatoms’ selection. (a)** The fitting and extraction process for a representative metaatom with length 560 nm and width 285 nm. **(b)** The filtered data points with the selection criteria. The red arrow shows the presentative metaatom shown in **(a)**.

**Fig. S4a** shows the phase plot as a function of frequency for a representative metaatom. The phase curve is nearly linear within the operating frequency range. The filtered data points with the selection criteria are shown in **Fig. S4b**. Each coordinate value represents a set of data that is ready to use. This conversion makes it easy to find the correspondingly metaatoms that cover the largest group delay to realize achromatic focusing.

1. ***Achromatic annular focusing with metaatoms***

In this work, adopting metaatoms that meet geometric phase conditions provides an easy way to control the additional phase by rotating the structure. The phase of the center wavelength can be arbitrarily changed while the dispersion characteristics remain unchanged. When selecting achromatic metaatoms, we only need to consider whether the metaatoms satisfy geometric phase conditions and have linear dispersion slopes. The geometric phase metaatoms have more degrees of freedom for phase modulations than those of traditional unpolarized structures.


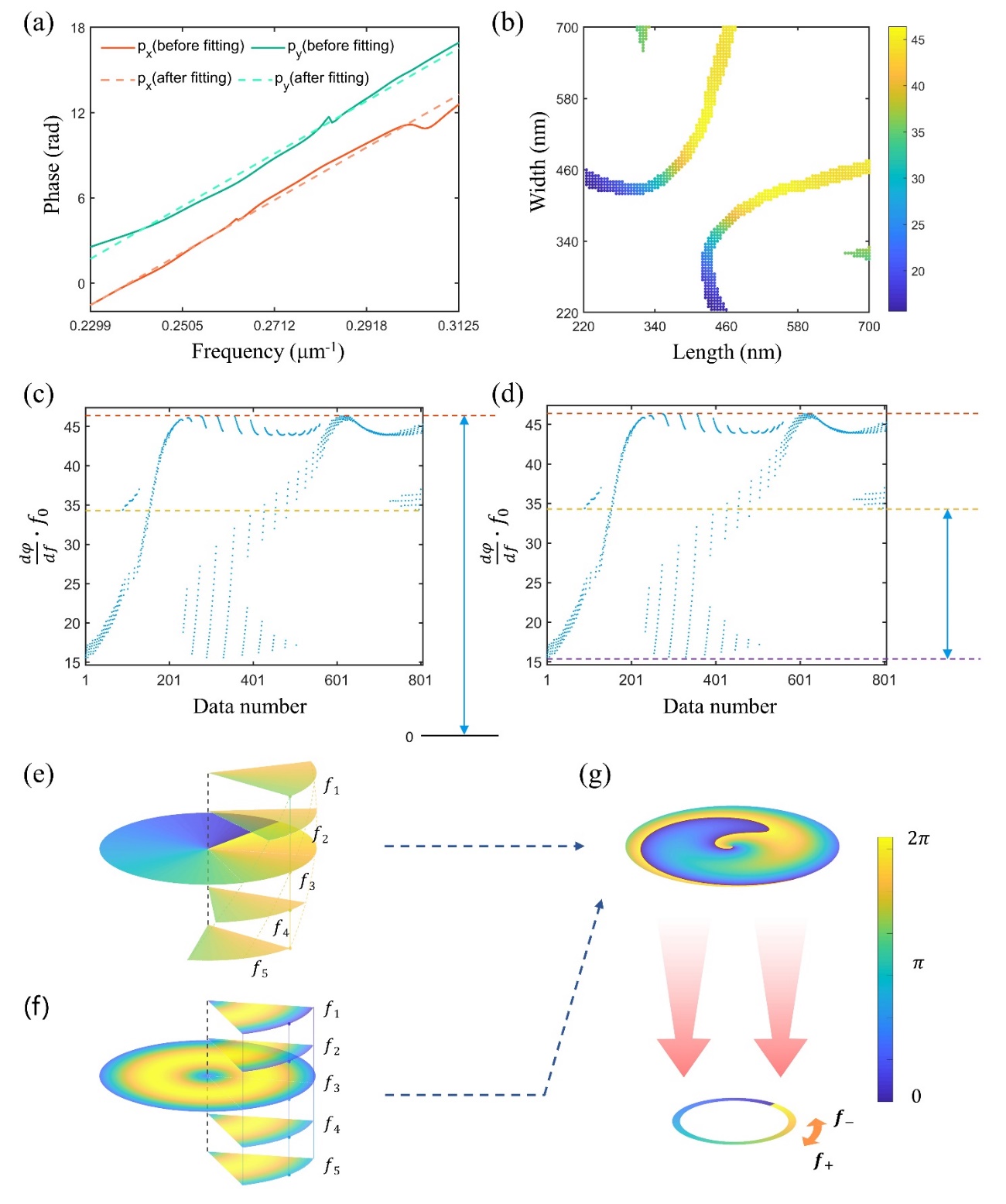


**Fig. S5 Schematic of achromatic focusing of annular vortices. (a)** A representative all-Si birefringent metaatom that satisfies the geometric phase condition within a broad bandwidth. **(b)** The metaatom's detailed width and length meet the achromatic geometric phase condition. Color bar represents the group delay. **(c)** Vortex dispersion selection for the metadevice design. The data number represents the metaatom number with filtering criteria in achromatic design. **(d)** Achromatic dispersion selection for metasurface design. **(e)** Vortex phases distributions at different frequencies. **(f)** The phase distributions as a function of frequency for annular focusing. **(g)** The phase profile combines the vortex phase in **(e)** and the annular focusing phase in **(f)**. It has rotating phase distributions as incident frequency changes.

**Fig. S5a** shows the phase dependence on the incident frequency for a representative metaatom. The phase dispersion of the metaatoms is also illustrated and fitted with dashed lines. Two parallel lines with the same group delay ensure that the geometric phase condition is satisfied within the whole bandwidth. The all-Si birefringent metaatoms are chosen to satisfy the achromatic geometric phase condition. To construct the required phase profiles, we first build the database by sweeping the length and width. Then, the metaatoms satisfying the geometric phase conditions over the operation bandwidth are respectively picked up as shown in **Figs. S5b-d**. The vertical double-headed arrow in **Fig. S5c** indicates the absolute position of the group delay from the benchmark, which determines the rotation speed of the phase profiles. The vertical arrow in **Fig. S5d** represents the phase dispersion coverage and determines the largest phase dispersion control range.

**Fig. S5e** represents the vortex phase and the phase dispersion. The phase plane rotates with frequency, and the rotation speed is related to the slope of the metaatom’s dispersion. **Fig. S5f** shows the phase requirements for achromatic annular focusing. The phase profiles at different frequencies are illustrated. The width of the annulus gets narrower as the frequency increases. **Fig. S5g** shows the phase profile of the superposition of the two phase profiles in **e** and **f**. The overall effect is to focus light into an annulus on the same focal plane at different frequencies. The dispersion control and achromatic focusing in **Figs. S5e** and **f** guarantee the annular focusing with the same radius over the whole operation bandwidth and the angle-resolved distributions at different frequencies. This is why we use achromatic design to enhance the device's performance.

1. ***Analytical expression of the rotation angle on the annulus***

The effect of group delay coverage difference on the rotation angle range can be expressed as:

$\theta_{A}\left( f \right)=\frac{k_{A}*\left( f-f_{0} \right)*360}{2\pi}$ (s10)

$\theta_{B}\left( f \right)=\frac{k_{B}*\left( f-f_{0} \right)*360}{2\pi}$ (s11)

where $\theta_{A}$ and $\theta_{B}$ are the rotation angles of Group A and Group B, respectively. $k_{A}$, and $k_{B}$ represent the benchmark of group delay of Group A and Group B. $f_{0}$ represents the center frequency, $f$ is the varying frequency. $k_{A}$ and $k_{B}$ are obtained through the phase fitting curves of the metaatoms, which are expressed as: $k_{n}=\frac{d\varphi\left( f \right)}{df}$. $\varphi$ is the phase after linear fitting.

According to **Eq. s10**, the rotation speed of the two vortices with respect to frequency can be obtained as follows:

$V_{fA}=\frac{d\theta_{A}}{df}=\frac{k_{A}*360}{2\pi}$ (s12a)

$V_{fB}=\frac{d\theta_{B}}{df}=\frac{k_{B}*360}{2\pi}$ (s12b)

The emerging in-pair interference spots on the annulus rotate as the frequency. The angle of enhanced interference spots can be written as:

$\theta_{x}\left( f \right)=\frac{-k_{A}*\left( f-f_{0} \right)+k_{B}*\left( f-f_{0} \right)}{2}=\frac{\left( -k_{A}+k_{B} \right)*\left( f-f_{0} \right)}{2}$ (s13)

With this equation, the analytical angle of spots shows a linear dependence on the frequency can be determined. It also allows the identification of the driving frequency by simply reading the angle of interference spots. When more dimensions such as frequency and polarization are incorporated, more interference annulus needs to be added.

## Note 3. The adoption of vortices carrier

The underlying physics of the photonic slide rule in our work is manipulating the dispersions difference. The adoption of vortex phases provides an excellent carrier to convert the dimensions of incident photons to the angle-resolved interference pattern in the far-field. As an alternative, we can also convert the frequency change into the interference intensity difference through the dispersion difference without the vortices. However, this law has no advantage over other frequency representation methods. To demonstrate this, in the following, we validate and emphasize the necessity of introducing the vortex phases with a comparison of other forms. Besides, the influence of the topological charge number on the output pattern is also investigated.

- 1. ***Situations of homogenous phases***


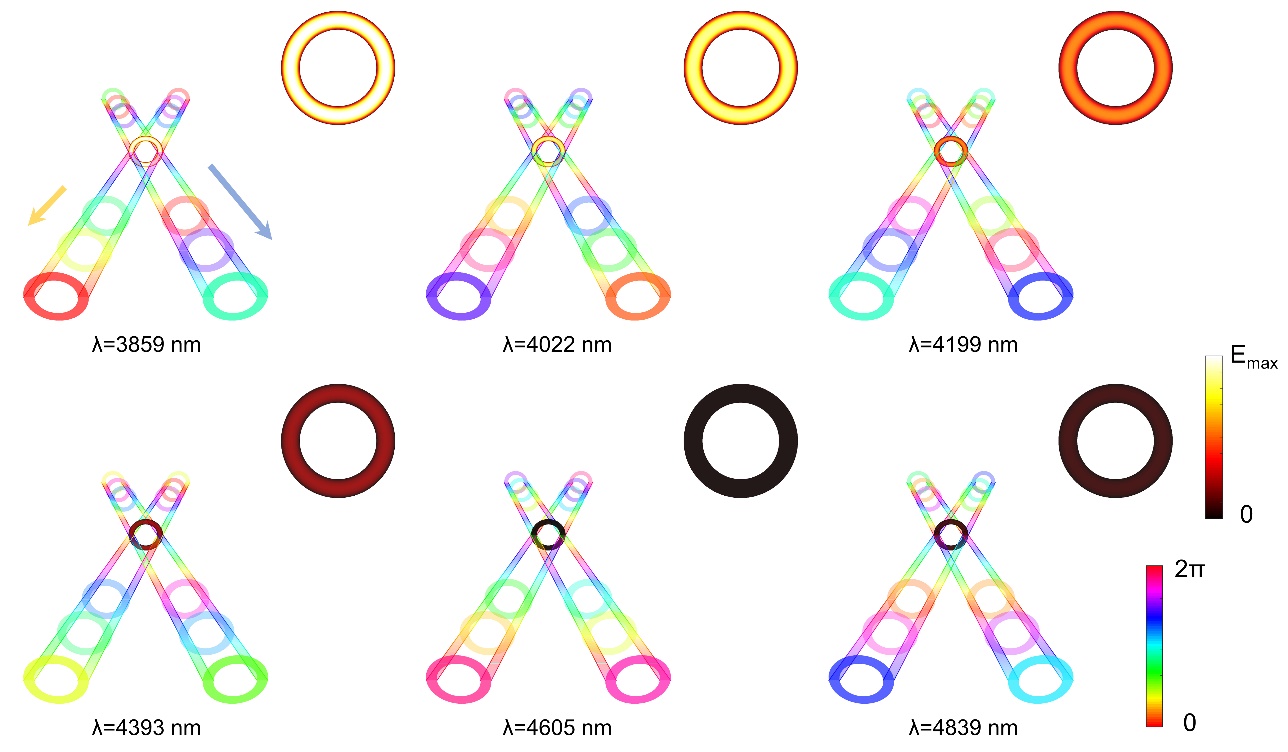


**Fig. S6 The interfering processes and output of the photonic slide rule with homogenous phase carrier at different frequencies. The color bars on the right correspond to the far-field intensity and evolving phase, respectively.**

As shown in **Fig. S6**, with homogenous phase carrier rather than the vortices, the two phase profiles generated by Groups A and B are still dependent on the frequency, but the output on the focal plane only have intensity differences. This is easy to interfere with but cannot be recognized visually. The lower-left corner of each subgraph illustrates the schematic evaluation process of phases along the propagation direction. The upper right corner shows the enlarged view of interfered intensity patterns. Among them, Group A was set to have a uniform phase variation of 4π, and Group B was set to have a uniform variation of 5.75π. The average intensities on the interference rings in the figure are 1, 0.872, 0.552, 0.206, 0.011, and 0.067, respectively.

- 1. ***Situations with vortex phases***

The two phase profiles evolve at different speeds for vortex phase carriers and interfere to generate angle-resolved intensity distributions on the focal plane for different frequencies, as shown in **Fig. S7**. The interfered pattern is a symmetric annulus. The strongest in-pair spots rotate with the incident frequency. Compared with those in **Fig. S6**, the angle-dependent spots are much easier to detect and recognize.


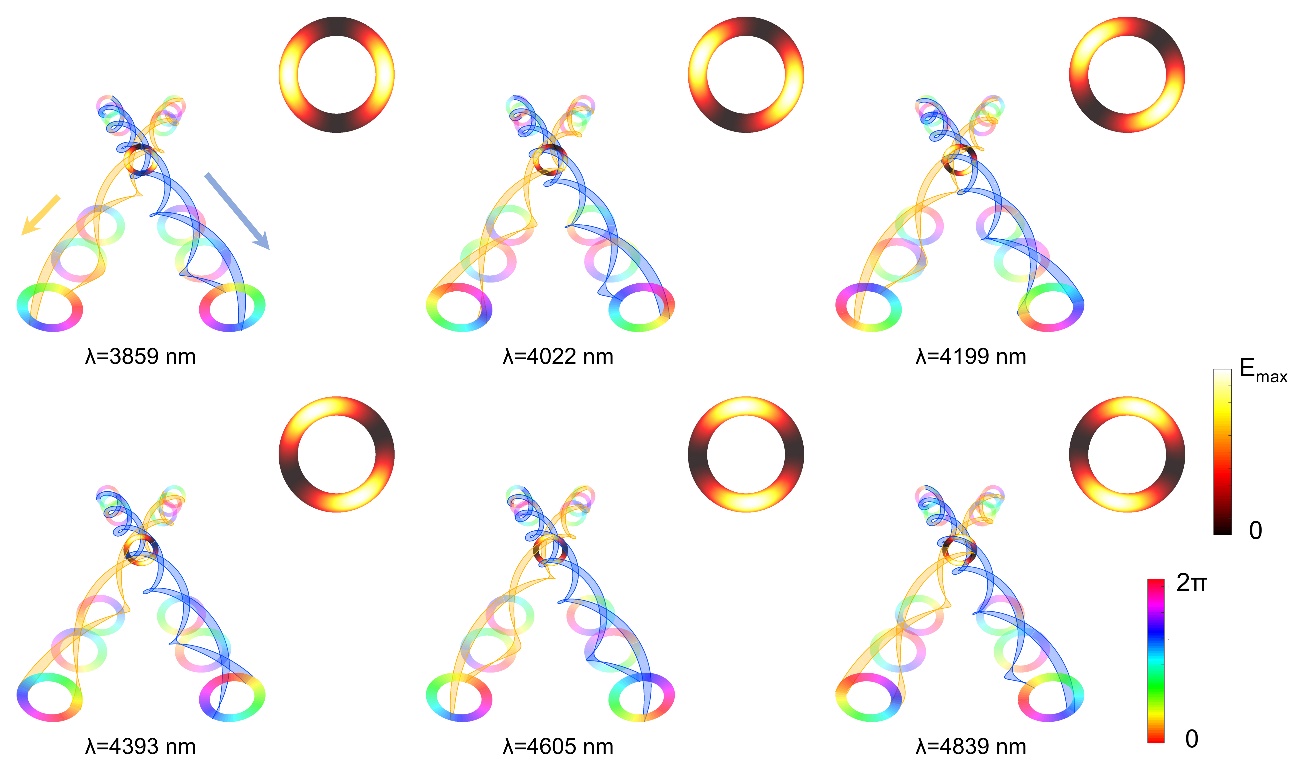


**Fig. S7 The interfering processes and output of the photonic slide rule with vortices as the carrier at different frequencies. The color bars on the right correspond to the far-field intensity and evolving phase, respectively.**

- 1. ***The influence on the selection of topological charge number***

For metadevices design in the main text, we choose topological charges +1 and -1 for the operating carriers. Its interaction process can be found in **Fig. S8**. The overall phases at the center wavelength can be expressed as φ_A_ and φ_B_, which are the addition phase and the initial phase. It should be noted that the initial phase is dependent on the frequency and can be adjusted. The superposition of vortices A and B would lead to the annular distributed intensities in the far-field. It’s worth mentioning that once the initial phase difference is fixed at the center wavelength, the starting angle in the far-field is determined. With the delicate dispersion manipulation, the device can be controlled to perform as those in the main text to characterize the dimensions of incident photons.


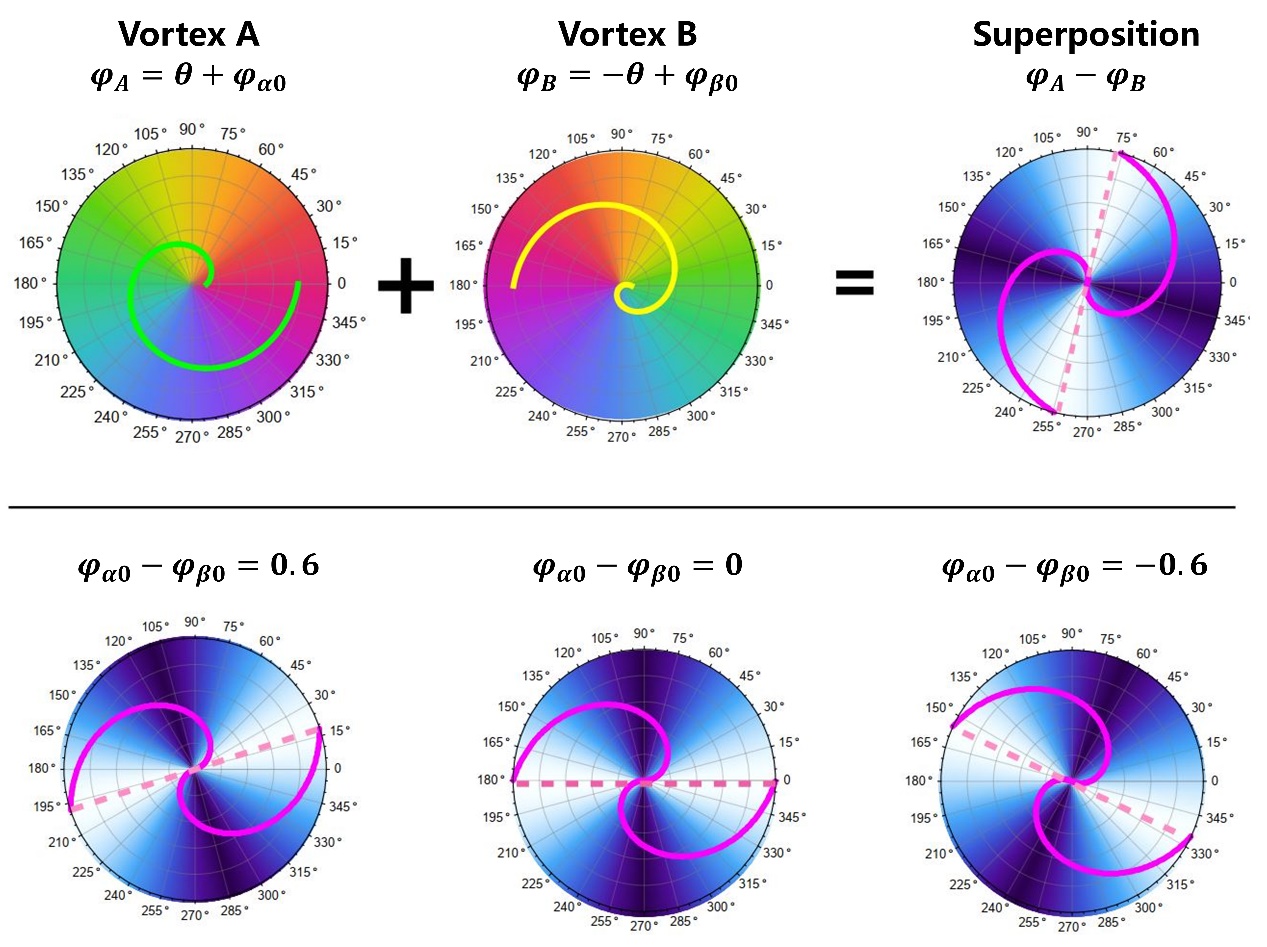


**Fig. S8 Schematic diagram of two vortices’ superposition. The overall interference is determined by the phase difference between the two phase profiles.**

Besides, it is worth noting that although we choose topological charges +1 and -1 for demonstration in the main text, the selection of topological charges is not limited. To prove this, cases for different topological charges are shown in **Fig. S9**. The subtraction value of the selected topological charges number determines the number of lobes. Theoretically, more lobes lead to more degrees of freedom for characterization. The energy allocated to each lobe would decrease.


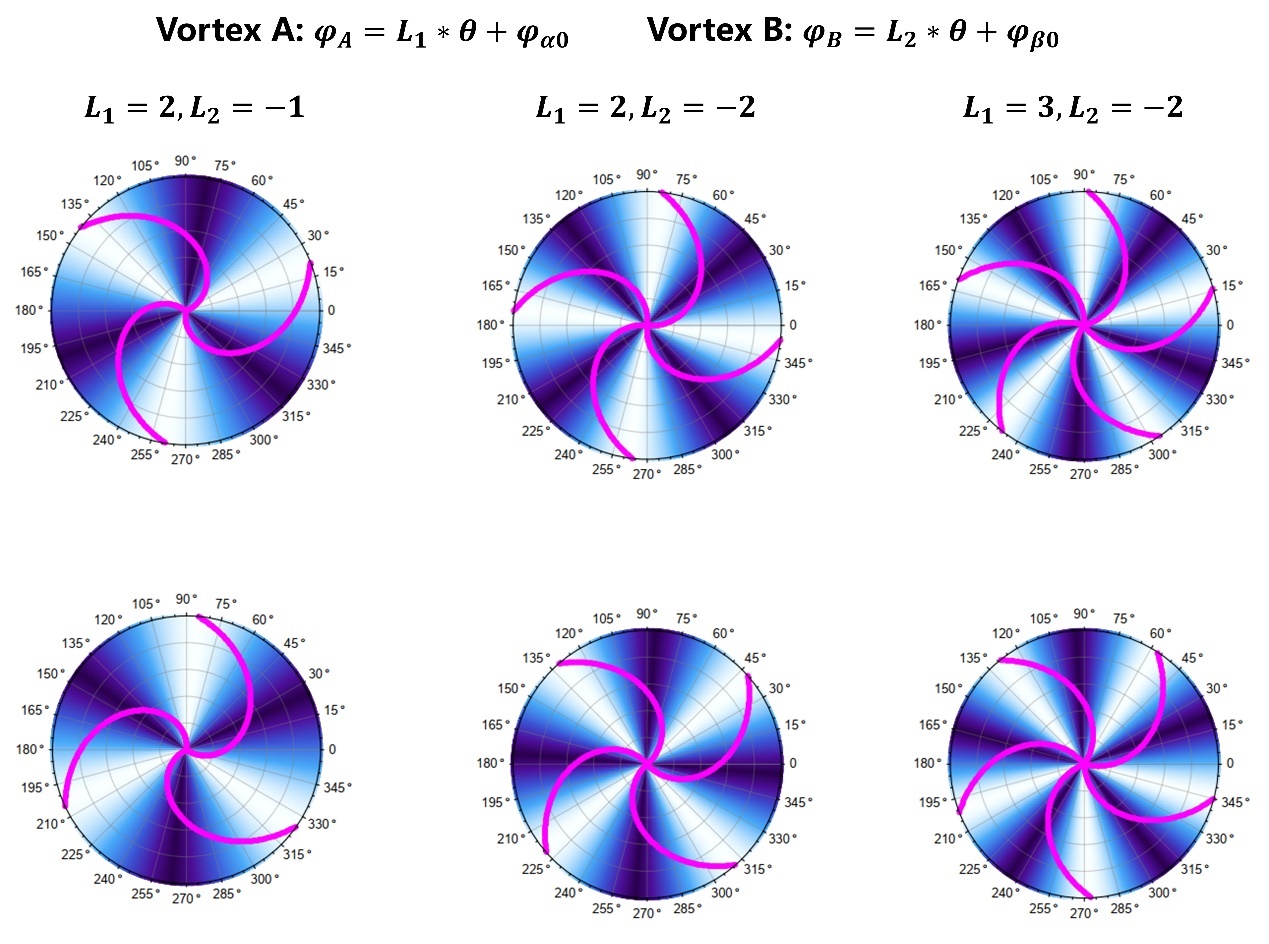


**Fig. S9 The cases for different topological charge values. The subtraction value of the topological charge number determines the number of lobes.**

## Note 4. Simulation results for frequency characterization

We also conducted the full-wave simulation to verify the metadevice performance. The size of the metasurface in simulation is 200 μm, considering the restriction of memory and computing time with our calculation cluster. The working distance is 160 μm. We keep the focal length ratio and the metasurface's size a constant in simulation. The simulation result is shown in **Fig. S10**. The interfered patterns operate at a satisfactory level compared with those measured in the experiment. A larger size would improve the performance.


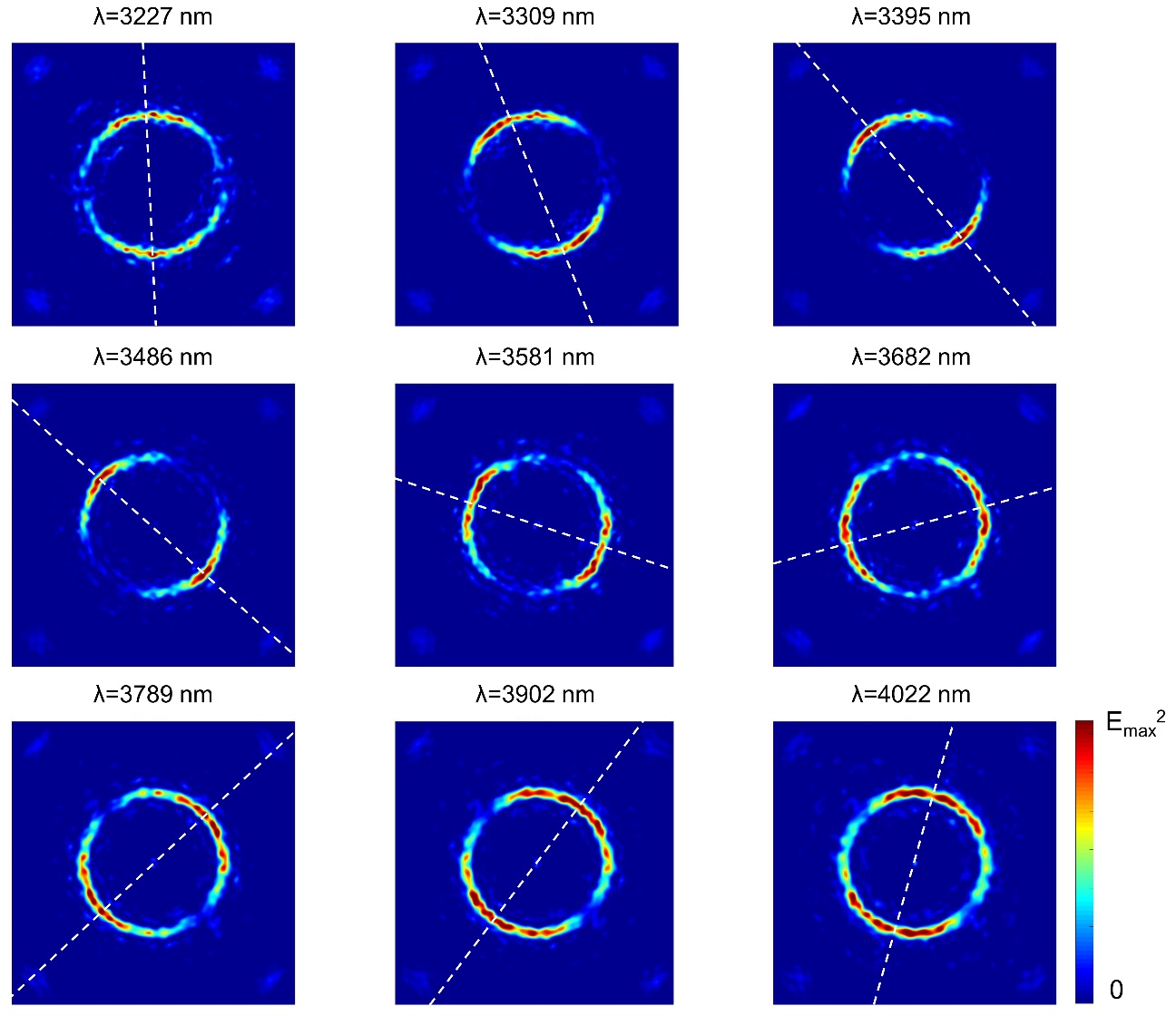


**Fig. S10 Simulation results of the photonic slide rule for frequency characterization. Intensity distributions at different wavelengths are illustrated.**

## Note 5. Characterization of elliptical polarization state

**
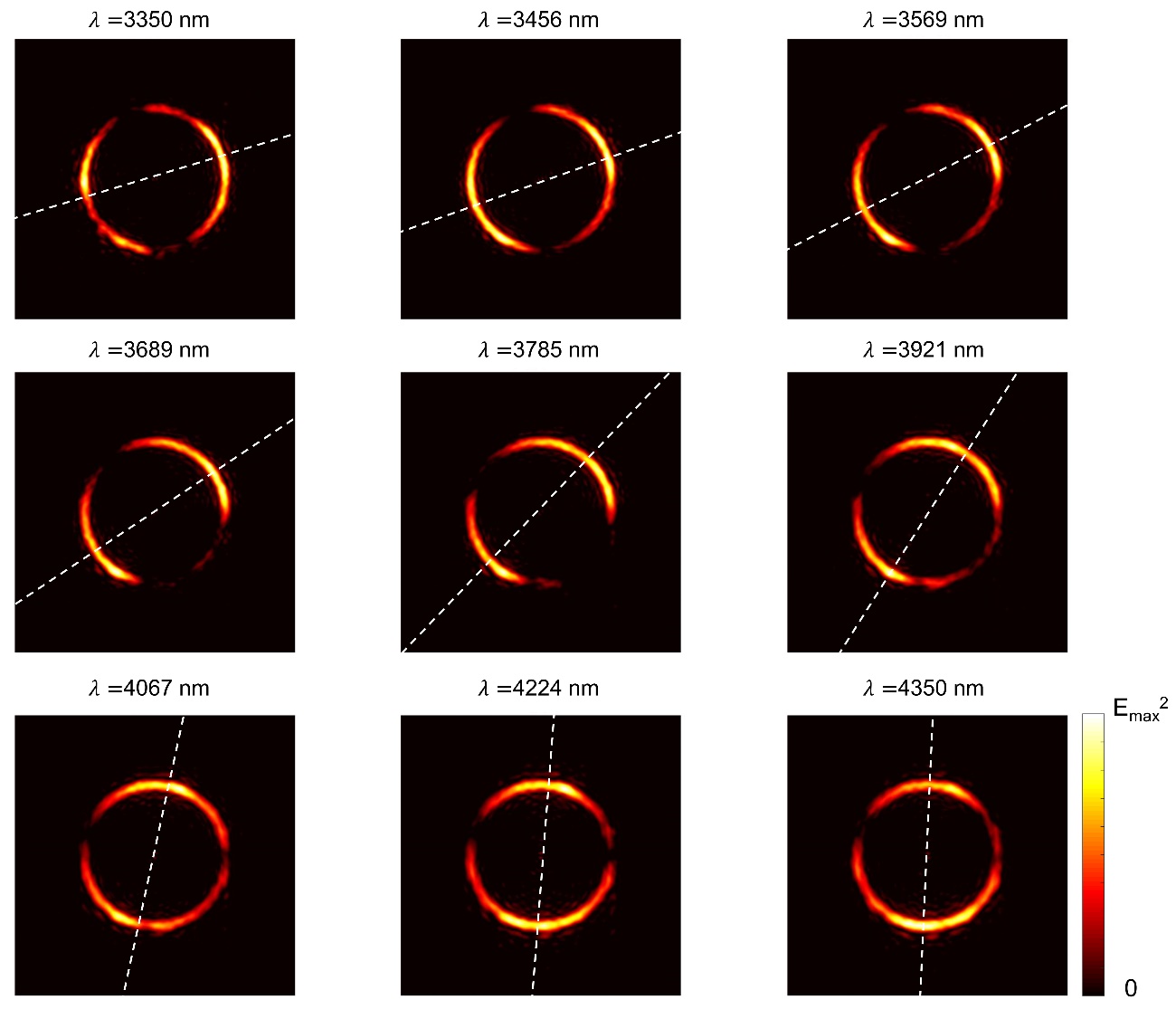
**

**Fig. S11 Full-wave simulation results for the characterization of elliptic polarization state at different wavelengths. The ellipsoid angle is π****/6.**

In the main text, we demonstrate the circular polarizations characterization with the photonic slide rule. Actually, the photonic slide rule can be further extended to characterize full polarization states. The all-Si metaatoms database can support the design of any polarization state resolving. To demonstrate this, we choose the most general case-elliptical polarization state ellipticity angle π/6 as an example. The simulation results can be found in **Fig. S11**. The numerical patterns confirm our design. The full polarization characterization can be fulfilled with another annulus in the far-field with this ability. As to the determination of the azimuthal angle, the third annulus, which operates based on another polarization basis which is not the circular one, is required. In this way, we have three focusing annuluses and corresponding maximum intensities to implement the unique determination of any polarization state on the Poincare sphere. The metadevice we designed can resolve any polarization state while maintaining the wavelength characterization function.
